# Otilonium Bromide treatment prevents nitrergic functional and morphological changes caused by chronic stress in the distal colon of a rat IBS model

**DOI:** 10.1111/jcmm.16710

**Published:** 2021-06-09

**Authors:** Chiara Traini, Eglantina Idrizaj, Rachele Garella, Maria‐Simonetta Faussone‐Pellegrini, Maria Caterina Baccari, Maria Giuliana Vannucchi

**Affiliations:** ^1^ Research Unit of Histology and Embryology Department of Experimental and Clinical Medicine University of Florence Florence Italy; ^2^ Section of Physiological Sciences Department of Experimental and Clinical Medicine University of Florence Florence Italy

**Keywords:** corticotropin‐releasing factor, faecal pellet, giant contractions, inducible nitric oxide synthase, irritable bowel syndrome, neuronal nitric oxide synthase, N^G^‐nitro‐L‐arginine, protein gene product 9.5, repeated water avoidance stress, tetrodotoxin

## Abstract

Irritable bowel syndrome (IBS) is a highly prevalent gastrointestinal disorder characterized by periods of remission and exacerbation. Among the risk factors to develop IBS, psychosocial stress is widely acknowledged. The water avoidance stress repeatedly applied (rWAS) is considered effective to study IBS etio‐pathogenesis. Otilonium bromide (OB), a drug with multiple mechanisms of action, is largely used to treat IBS patients. Orally administered, it concentrates in the large bowel and significantly ameliorates the IBS symptomatology. Presently, we tested whether rWAS rats developed neuro‐muscular abnormalities in the distal colon and whether OB treatment prevented them. The investigation was focussed on the nitrergic neurotransmission by combining functional and morphological methodologies. The results confirm rWAS as reliable animal model to investigate the cellular mechanisms responsible for IBS: exposure to one‐hour psychosocial stress for 10 days depressed muscle contractility and increased iNOS expression in myenteric neurons. OB treatment counteracted these effects. We hypothesize that these effects are due to the corticotropin‐releasing factor (CRF) release, the main mediator of the psychosocial stress, followed by a CRF1receptor activation. OB, that was shown to prevent CRF1r activation, reasonably interrupted the cascade events that bring to the mechanical and immunohistochemical changes affecting rWAS rat colon.

## 1 INTRODUCTION

Irritable bowel syndrome (IBS) is a highly prevalent gastrointestinal disorder^1^ whose symptoms (abdominal painful contraction, discomfort, alteration of bowel habits) are mainly referred to the large bowel.

IBS is considered a chronic multifactorial disease^2^ characterized by alternating periods of remission and of symptom exacerbation^3^ whose aetiology is still unknown. Among the risk factors to develop or re‐exacerbate the symptomatology, psychosocial stressing events are the most widely acknowledged.^4^, ^5^ Remarkably, up to 80% of IBS patients experiences comorbid behavioural disorders, such as anxiety or depression.^6^ The correlation between IBS and psychosocial stress strengthens the hypothesis of an enteric nervous system (ENS) engagement in the disease pathogenesis, likely, through the gut‐brain axis.^7^, ^8^, ^9^ Thus, although a modification of the ENS transmission in IBS patients is convincible, it has been poorly investigated in human due to the difficulty in obtaining gut full‐thickness specimens.^10^ Finally, although IBS is commonly listed as a functional disorder, presence of mucosal barrier alterations, low‐grade inflammation, brain‐gut axis dysfunction and dysbiosis are evidence for an IBS organic origin.^11^

Based on the well‐established importance of the psychosocial stressors in IBS^12^ and the difficulty in obtaining full‐thickness human specimens from these patients, the researchers focussed their attention on obtaining animal models that mimicked this disease. Several animal models have been generated^13^, ^14^, ^15^, ^16^, ^17^, ^18^, ^19^ but few of them adequately reproduce a condition of chronic psychosocial stress; among them, the water avoidance stress (WAS) applied repeatedly, (r)WAS, is considered one of the most effective for this purpose.^5^, ^20^, ^21^ Employed in the adequate rat strain (Wistar rats), rWAS creates a context in which the animal simultaneously experiences two different types of stress conditions: isolation and immobility, and after ten days of repeated exposures, develops chronic enteric hyperalgesia, increased faecal pellet output and a mild inflammation in the mucosa as suggested by the presence of polymorphonuclear cells aggregations, increased mast cells number and IL‐1 and INF‐γ cytokine expression.^20^

One of the most common and effective drugs for IBS is otilonium bromide (OB) a quaternary ammonium derivative that, once orally administered, concentrates mainly in the large bowel.^22^, ^23^ In IBS patients, OB dismisses abdominal pain and visceral hypersensitivity, quietens the abnormal colonic contractions and normalizes intestinal transit.^22^, ^23^ From the pharmacodynamics point of view, OB is a broad‐spectrum drug targeting different cells types, such as the smooth muscle cells and the neurons,^14^, ^24^, ^25^, ^26^ and conditioning the microbiota population.^27^ All these properties make OB an interesting drug and the comprehension of the cellular mechanisms responsible for its effectiveness in ameliorating the IBS symptoms might help in understanding the disease pathogenesis.

Based on these data, we tested whether rWAS rats, as a reliable model for IBS, develop colonic neuro‐muscular abnormalities and whether OB treatment prevents them. These objectives were achieved by combining functional and morphological studies mainly focussed on the involvement of the inhibitory nitrergic neurotransmission.

## 2 MATERIALS AND METHODS

### 2.1 Animals

Male Wistar rats weighting 150‐200 g were purchased from Charles River Laboratories Italia srl (Lecco, Italy) and housed 2‐3 per cage at CeSAL (Department of NEUROFARBA, UNIFI, Italy) under standardized temperature and humidity, with 12/12 h light/dark cycle and free access to food and water. All procedures were carried out in accordance with the European guidelines for care and use of laboratory animals (Directive 2010/63/UE) and approved by the Italian Ministry of Health (code: 916/16). The rats were housed in the room adjacent to the testing one and randomly divided in five groups:

1. Rats not exposed to stress or pharmacological treatment (Ctrl).
2. Rats subjected to repeated water avoidance stress (rWAS), for 10 consecutive days.
3. Rats experiencing the stress environment, without being subjected to rWAS (Sham).
4. Rats treated orally with OB during the period of stress application (rWAS+OB).
5. Rats treated orally with OB for 10 days, not subjected to stress (OB).

Each rat was weighed every 2 days to assess the weight gain.

### 2.2 Repeated Water Avoidance Stress (rWAS)

Repeated Water Avoidance Stress was applied as reported in Bradesi et al.^20^ In brief, the test apparatus consisted of a plexiglas tank (45 cm length, 25 cm width, 25 cm height), with a polygonal platform (10 cm length, 8 cm width, 8 cm height) fixed to the centre of the tank floor. The tank was filled with freshwater (25°C) up to 1 cm above the top of the platform and placed in the centre of a testing room. The water was dyed using a non‐toxic dark colour and changed before each section to remove the smell of the previous rat and to collect the faecal pellets. The animals were placed on the platform for 1 hour/d for 10 consecutive days between 9:00 AM and 02:00 pm, in accordance with the circadian rhythm. Sham rats were placed on the platform in the waterless tank for 1 hour daily for 10 consecutive days.

### 2.3 Faecal pellets

Faecal pellets collected in the tank were counted, stored in separated bins (one for each animal) and exposed to the same conditions of temperature and humidity for 24 hours to allow dehydration. Then, the pellets were weighed.

### 2.4 Otilonium Bromide (**OB)**

Otilonium Bromide (10 mg/kg/d) was added to the drinking water from the day before the start of the rWAS, and its concentration was adjusted every 2 days based on body weight gain and water intake. Drug intake was checked by measuring residual water in the bottle every 2 days.

### 2.5 Elevator plus maze (EPM) behavioural test

Elevator Plus Maze (EPM) behavioural test induces a conflict in the rats between their aversion to open spaces and heights and the instinct to explore new environments. It consists of a cross‐shaped apparatus made by two closed arms (zone 1) and two open arms (zone 2), lifted 70 cm from the floor. The rats were placed in the centre of the cross and left free to explore the apparatus for 5 minutes. EPM was applied the day before the start of rWAS application (Day‐1) and the day after the end of the stress period (Day 11).

### 2.6 Tissue sampling

At Day 11, the rats were anaesthetized and killed. The abdomen opened and the colon rapidly removed. The distal colon was divided in two segments: one for the functional experiments, the other for the morphological and biomolecular studies.

### 2.7 Functional experiments

As previously,^28^ two full‐thickness circular muscle strips (0.4 ± 0.1 cm) were dissected from each colon segment, mounted in 5 mL organ baths containing Krebs‐Henseleit solution composed by (in mmol/L): 118NaCl, 4.7KCl, 1.2 MgSO_4_, 1.2KH_2_PO_4_, 25NaHCO_3_, 2.5CaCl_2_ and 10glucose, pH7.4 and bubbled with 95% O_2_‐5% CO_2_. Temperature was maintained within a range of 37 ± 0.5°C. One end of each strip was tied to a platinum rod while the other was connected to a force displacement transducer (FT03; Grass Instrument) by a silk thread for continuous recording of isometric tension. The transducer was coupled to a polygraph (7K; Grass Instrument). Strips equilibrated for 1 hour under an initial load of 1 g. During this period, the preparations underwent repeated and prolonged washes with Krebs‐Henseleit solution to prevent accumulation of metabolites in the organ baths.

The following drugs were used: the nerve blocker tetrodotoxin (TTX, 1 × 10^‐6^ M), the NOS inhibitor N^G^‐nitro‐L‐arginine (L‐NNA, 2 × 10^‐4^ M) and the muscarinic receptors agonist methacholine (2 × 10^‐6^ M). Drug concentrations were those previously used in rodent gastrointestinal preparations and proved to be effective.^29^ All drugs were obtained from Sigma. Solutions were freshly prepared, except for TTX, for which a stock solution was kept stored at −20°C.

### 2.8 Morphological studies

Full‐thickness segments of distal colon were fixed in 4% paraformaldehyde in 0.1 M phosphate‐buffered saline (PBS, pH 7.4) over night (ON) at 4°C, dehydrated, cleared and embedded in paraffin with the cut section transversal to the longitudinal axis. Then, 5 µm thick full‐thickness sections were cut using a rotary microtome (MR2, Boeckeler Instruments Inc Tucson) and collected on slides.

The sections were deparaffinized and rehydrated as usual. For light microscopy, different sections were processed for haematoxylin‐eosin (HE), Toluidine Blue or Periodic Acid and Schiff (PAS) staining, using routine methods. For immunohistochemistry (IHC), the sections were treated for 20 minutes at 90‐92°C in tris buffer (10 mmol/L) with EDTA (1 mmol/L, pH 9.0), followed by cooling to RT for antigen retrieval. Then, the sections were washed in PBS and blocked for 20 minutes at RT with 1.5% bovine serum albumin (BSA, Applichem) in PBS. To evaluate the percentage of neuronal nitric oxide synthase (nNOS)‐ and inducible nitric oxide synthase (iNOS)‐immunoreactive (IR) neurons respect to the total neurons, labelled with the PGP9.5 pan‐neuronal marker, only polyclonal antibodies were used since the monoclonal nNOS antibody labels a lower percentage of neurons respect the polyclonal nNOS.^30^ Thus, to recognize the same ganglia and count the same neurons sequential sections were collected on slides (4 sections/slide, 2 slides/animal) in two separate areas, one area containing the first and the third section, the other area containing the second and the fourth section and the two sections of one area were incubated with PGP9.5 antibody and the two sections of the neighbour area with nNOS or iNOS antibodies. The primary antibodies (Table S1) diluted in 1.5% of BSA in PBS were applied ON at 4°C. The day after, the sections were washed in PBS and incubated for 2 hours at RT in the dark with appropriate fluorochrome‐conjugated (Alexa Fluor 488) secondary antibodies (Table S1) diluted in PBS. The sections were washed in PBS, incubated 10 minutes with Hoechst 33 342, a nuclei marker (20 μg/mL; Sigma) dissolved in PBS, washed in distilled water and mounted in an aqueous medium (Immuno‐Mount, Thermo Scientific, Rockford). To verify whether the same neurons express the two NOS isoforms, the polyclonal iNOS and the monoclonal nNOS antibody (Table S1) were applied sequentially, each incubated ON at 4°C and following the procedure described above for each antibody. To identify the mast cells in the mucosa, the c‐kit antibody (Table S1) was diluted in 1.5% of BSA in PBS and applied ON at 4°C. The day after, the sections were washed in PBS and incubated for 2 hours at RT in the dark with appropriate fluorochrome‐conjugated (Alexa Fluor 488) secondary antibody (Table S1) in PBS. The sections were washed in PBS and mounted in an aqueous medium (Immuno‐Mount, Thermo Scientific). Negative controls were performed omitting the primary antibody to exclude the presence of non‐specific immunofluorescence staining.

Immunoreactivity was observed under the Olympus BX63 fluorescence microscope (Olympus), and the signal was obtained using 488‐ and 370‐nm excitation wavelength for the green and blue fluorescent labels, respectively, and the photographs were taken via the associated imaging system (CellSens Dimension Imaging Software, Olympus).

### 2.9 Western blotting (WB)

Colon samples were homogenized in ice‐cold lysis buffer (10 mmol/L Tris/HCl pH 7.4, 10 mmol/L NaCl, 1.5 mmol/L MgCl2, 2 mmol/L Na2EDTA, 1 mmol/L phenylmethylsulfonyl fluoride (PMSF), 1% Triton X‐100) supplemented with Sigma fast Protease Inhibitor cocktail tables (Sigma‐Aldrich). Upon centrifugation (13.000 *g*, 30 minutes, 4°C), the supernatants were collected, the total proteins measured spectrophotometrically using a BCA Protein Assay Reagent Kit (Pierce, Rockford), following the manufacturer instructions. 70 µg of total proteins and appropriate molecular‐weight markers (Bio‐Rad, Hercules) were electrophoresed by SDS‐PAGE (200 V, 45 minutes) using a denaturating 7.6% polyacrylamide gel and blotted (150 V, 1 hour) onto polyvinyl difluoride (PVDF) membranes (Invitrogen). After thorough washings in PBS containing 0.1% Tween (PBS‐T; Sigma‐Aldrich), the membranes were treated on rotary shaker with T‐PBS containing 5% BSA (Sigma‐Aldrich) at RT for 2 hours and incubated ON at 4°C with a rabbit nNOS antibody and with mouse or rabbit iNOS‐antibody (Table S1). The immune reaction products were revealed by incubating membranes with adequate peroxidase‐conjugated secondary antibodies (Table S1) for 1 hour at RT. Immunoreactivity was detected by chemiluminescence reaction. The WB runs were then stripped and immunostained with anti‐β‐actin or α‐tubulin (Table S1), as housekeeping proteins. Densitometric analysis of the bands was performed using ImageJ software (http://rsbweb.nih.gov/ij), and the values normalized to α‐tubulin bands.

### 2.10 Quantitative and statistical analysis

The animal weight and the faecal pellet number and weight were quantified and expressed as means ± SEM The percentage of the time spent on the platform (zone 2) or in the remaining space of the tank (zone 1) during rWAS procedure and the time spent plus the distance crossed by each rat in the zone 2 of EPM were directly recorded and quantified by the computer‐digitizing system (HVS Image) supplied in the test room; the results are expressed as mean% ± SEM

Amplitude of spontaneous contractile activity was expressed as absolute values (grams) or as percentage changes in respect to the own control (taken as 100%) for each animal group, following drugs addition to the bath medium. Results are given as ± SEM The number of muscle preparations is designated by *n* in the results.

The quantitative analysis of the neuronal immunoreactivity (4 sections/slide; 2 slides/animal) and of the mast cell number (2 sections/slide; 2 slides/animal) was done by two observers (CT; EI), blind to each other, along the entire section. Only the neurons containing the nucleus were included. The results were reported as percentage of the PGP9.5‐positive neurons for section ±SEM The number of animals used is reported as n in the legends. Statistical analysis was performed by means of paired or unpaired Student's *t* test or one‐way ANOVA followed by Newman‐Keuls post‐test. Values were considered significantly different with *P* < .05.

## 3 RESULTS

### 3.1 Rat body weight and water intake

**FIGURE 1:**
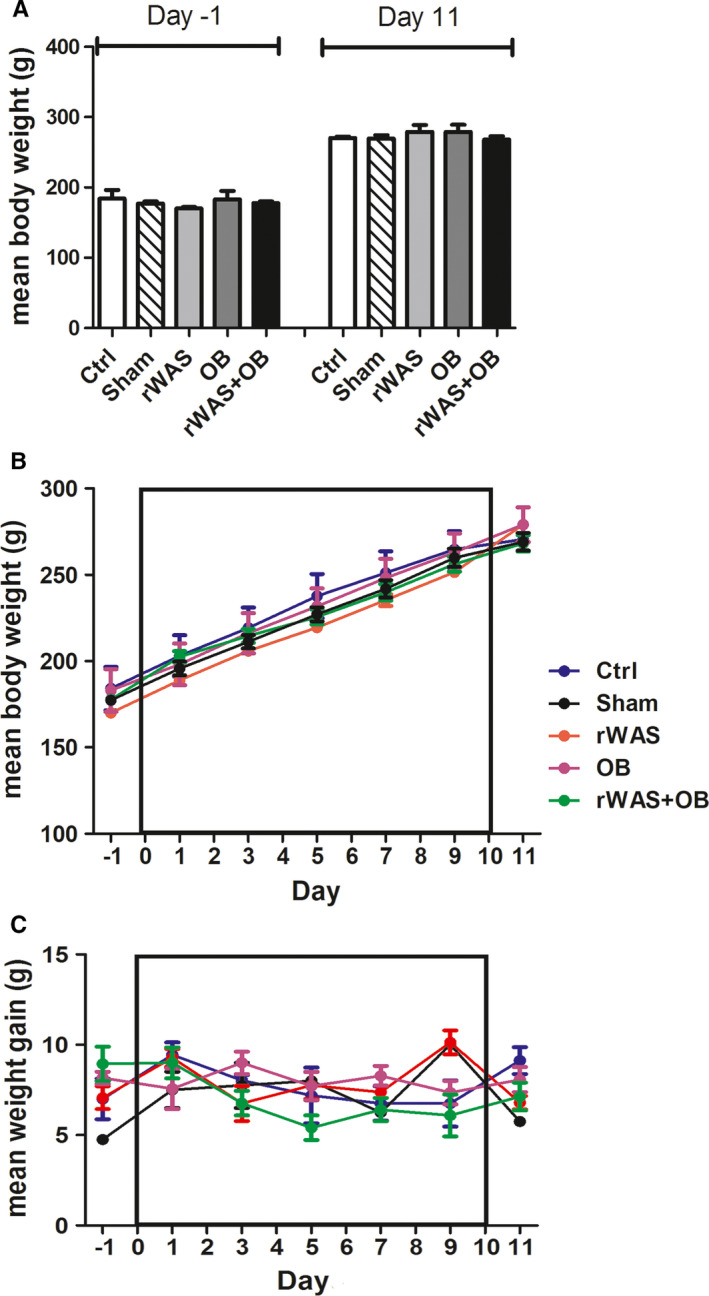
Weight gain. No significant difference was observed in the body weight among the experimental groups (A) either before the starting of rWAS application (Day −1) or the day after the last exposure to stress (Day 11). The increase of the body weight (B) and the daily weight gain (C) during the 10 d of stress application were relatively constant and similar among the groups. One‐way ANOVA followed by Newman‐Keuls post‐test, n = 7

At Day‐1, the rat body weights were not significantly different among the experimental groups (Ctrl: 184.1 ± 12.55 g; Sham: 177.3 ± 2.80 g; rWAS: 170.3 ± 1.82 g; OB:183 ± 13.31 g; rWAS+OB: 177.8 ± 2.56 g). During the treatment, the animals gained a similar increase in weight: mean value (expressed as g/die) was 7.7 ± 0.4 for Ctrl; 8.3 ± 0.4 for Sham; 7.5 ± 0.4 for rWAS; 8.0 ± 0.3 for OB and 7.7 ± 0.4 for rWAS+OB rats. Consequently, at Day 11, the body weight was not significantly different among the groups (Ctrl: 270.5 ± 1.8 g; Sham: 269 ± 5.1 g; rWAS: 279.0 ± 9.9 g; OB: 279.0 ± 10.1 g; rWAS+OB: 268.6 ± 4.7 g) (Figure 1).

The daily water intake was not different among Ctrl, Sham and rWAS groups (Ctrl: 40.34 ± 1.7 mL; Sham: 39.70 ± 2.0 mL; rWAS: 45.48 ± 2.4 mL) while, as expected, the bitter taste of OB dissolved in the drinking water caused a significant decrease (*P* < .005) of water intake in OB and rWAS+OB groups (OB: 32.32 ± 1.0 mL; rWAS+OB: 27.59 ± 1.4 mL) (Figure S1A).

### 3.2 Behavioural outcome of rWAS and EPM implementation

#### 3.2.1 Behavioural outcome

rWAS rats spent much of the time on the platform mainly motionless; occasionally, they turned around on the platform and, during the first days of stress application, jumped sometimes into the water and swam along the tank borders. It was estimated that the rWAS and rWAS+OB rats spent 85.65 ± 0.3% and 85.94 ± 1.7% of the hour on the platform (zone 2) and 14.35 ± 1.3% and 14.06 ± 1.7% into the water (zone 1), respectively. These results ensured the achievement of the immobility stress in the rats. Conversely, Sham rats preferred to stay at the bottom of empty tank (99.16 ± 0.9%) in the corners or along the borders; rarely, they climbed to the top of the platform (0.84 ± 0.29%) (Figure S1B).

#### 3.2.2 EPM implementation

**FIGURE 2:**
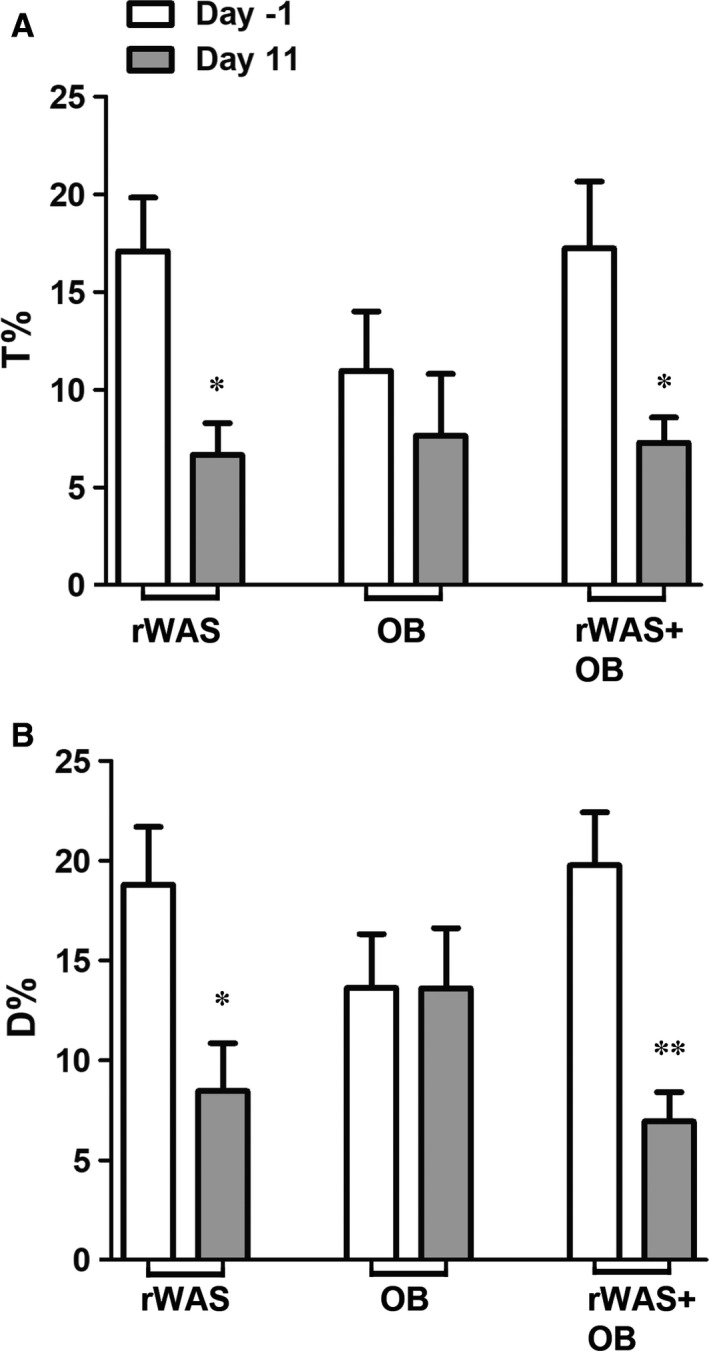
Elevated Plus Maze (EPM) test at baseline (Day‐1) and the day after the last rWAS application (Day 11) in rWAS, OB and rWAS+OB groups. The rat anxiety‐like behaviour was estimated as time spent to explore (A) and as the space explored (B) in the open arms of the maze. At Day‐1, the rat behaviour was not statistically different among the experimental groups (white columns; one‐way ANOVA of T%, *P* = .468; one‐way ANOVA of D%, *P* = .283; n = 7). At Day 11 (grey columns), the rat exploratory behaviour in the rWAS and rWAS+OB groups was significantly reduced respect to their own performances before the stress procedure (Paired two‐tailed Student *t* test, A: rWAS *P = .0129, rWAS+OB *P = .048; B: rWAS *P = .0294, rWAS+OB **P = .0065; n = 7*)*. D%, distance percentage; T%, time percentage

To set the baseline behaviour, the rat response to the EPM test was evaluated at Day‐1 and, to confirm the anxiety‐like behaviour produced by rWAS application, at Day 11 (Figure 2). At Day‐1, all the groups showed similar behaviours, while at Day 11, rWAS and rWAS+OB rats showed a significant reduction of the time spent to explore the open arms of the maze and of the walking respect to Day‐1 (Figure 2A,B).

### 3.3 Faecal pellet production

**FIGURE 3:**
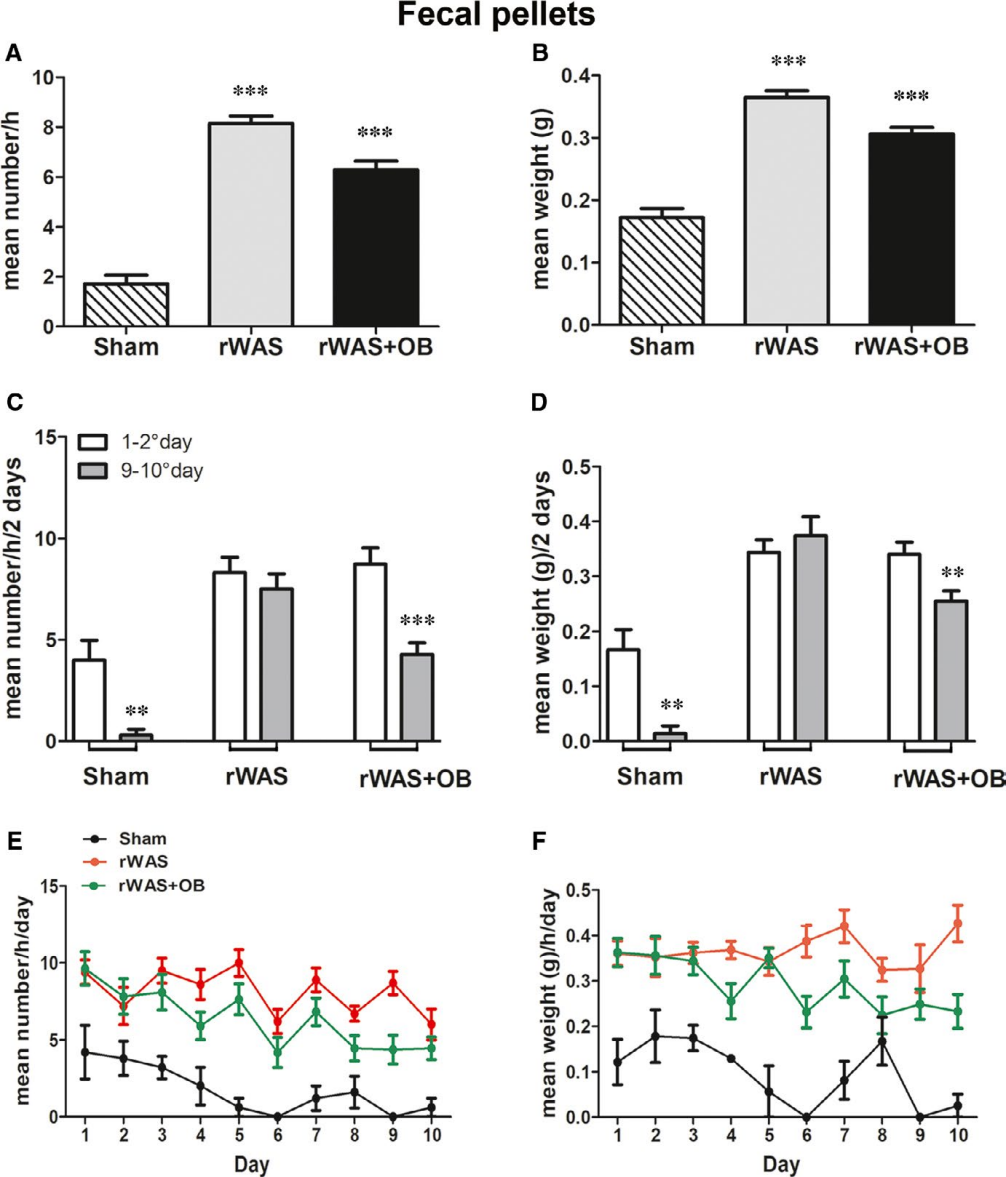
Faecal pellet production. The total mean number (A) and weight (B) of the faecal pellets was significantly higher in rWAS and rWAS+OB groups compared to Sham and between rWAS and rWAS+OB ones. One‐way ANOVA followed by Newman‐Keuls post‐test, ***P < .0005. The number and weight of the faecal pellets expressed as mean values for the first two and for last two days of stress application (C and D) significantly decreased in the Sham and in the rWAS+OB groups of rats but not in the rWAS one. (Paired two‐tailed Student *t* test, C: Sham **P = .0056, rWAS+OB ***P < .0001; D: Sham **P = .0058, rWAS+OB **P = .0062*)*. E and F, show the mean values per day of stress application of the faecal number (E) and faecal weight (F), n = 7

Faecal pellets were collected every day at the end of the one‐hour session for each rat exposed to the stress or for each Sham rat, counted and, after 24 hours of air exposition, weighed. The mean number and mean weight were significantly higher in the rWAS and rWAS+OB groups compared to Sham rats (Figure 3A,B). Notably, in the Sham and rWAS+OB groups, the production of faecal pellets was higher during the early days of stress application and decreased significantly with time (Figure 3C‐F) suggesting the appearance of environment habituation or confirming the drug efficacy, respectively, Conversely, in the rWAS rats, the faecal production did not change with time (Figure 3C‐F). The number and weight of the faecal production were evaluated also in controls and OB rats collecting the pellets from the cage during the ten experimental days. The results did not differ from those obtained in the Sham rats (data not shown).

### 3.4 Spontaneous mechanical activity

**FIGURE 4:**
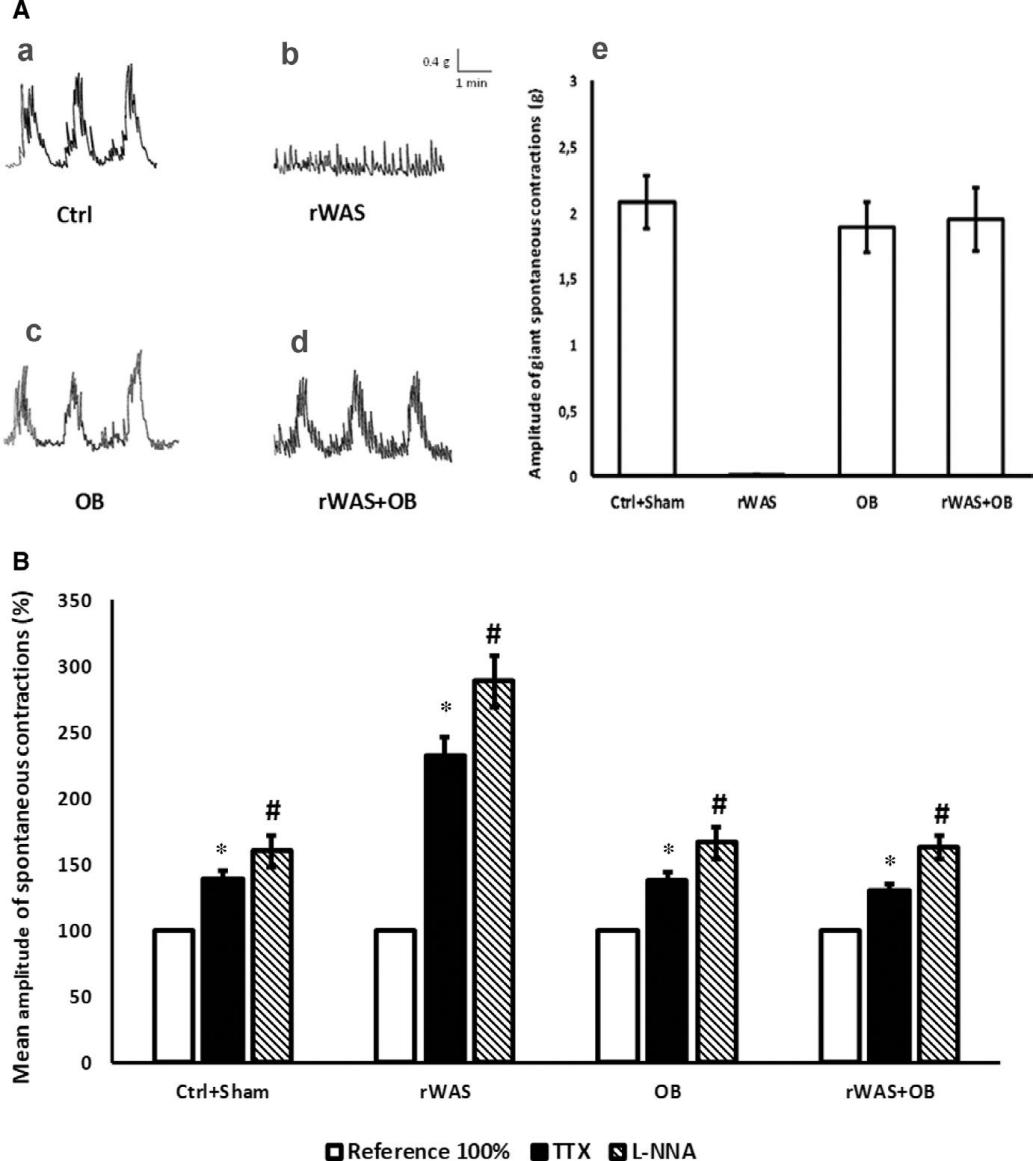
Spontaneous motility patterns recorded in preparations from the different animal groups: effects of TTX and L‐NNA on the mean amplitude of the spontaneous contractions. A, On the left: typical tracings showing the mechanical activity in preparations from the different animal groups: Ctrl (a), rWAS (b), OB (c) and rWAS+OB (d) rats. Preparations from Ctrl, OB and rWAS+OB rats exhibit high‐amplitude contractions (giant contractions, GCs) superimposed on small‐amplitude oscillations. Note the absence of the GCs in rWAS rats. On the right (e): bar chart showing the mean amplitude of the spontaneous GCs in preparations from the different animal groups. All values are means ± SEM of 8‐12 preparations. B, Bar chart showing the effects of TTX (1 × 10^‐6^ M) and L‐NNA (2 × 10^‐4^) on the mean amplitude of the spontaneous contractions in the strip preparations. The amplitude of contractions was measured 30 minutes following the addition of the drugs to the bath medium. The amplitude of spontaneous contractile activity is expressed as percentage increase in respect to the own control for each animal group, taken as 100%. Note that the percentage increase of the small spontaneous contractions induced by both TTX and L‐NNA in rWAS preparations is even major than that caused by the drugs on the GCs of all the other animal groups. All values are means ± SEM of 5‐6 preparations. *P < .05 vs its own control (taken as 100%); ^#^ *P* < .05 vs its own control (taken as 100%) and TTX (ANOVA and Newman‐Keuls post‐test)

Preparations from Ctrl (n = 20) rats exhibited high amplitude (mean amplitude 2.2 ± 0.22 g) contractions (giant contractions, GCs) superimposed on small amplitude (mean amplitude 0.12 ± 0.03 g) oscillations (Figure 4A). In a minority (25%) of recordings, one of the two strips dissected from the same colon showed only small rhythmic contractions. Therefore, the results reported below referred to the motility patterns recorded in most preparations. Preparations from Sham (n = 10) animals exhibited the same motility pattern (data not shown) and similar GCs amplitude values (mean amplitude 2 ± 0.19 g) of the Ctrl ones. Thus, the data were put and analysed together (Figure 4Ae).

In preparations from rWAS rats (n = 18), the GCs were never observed and the spontaneous motility pattern consisted only of small‐amplitude (0.5 ± 0.06 g) contractions (Figure 4Ab,e). Addition of methacholine to the bath medium, in strips from rWAS (n = 6) and Ctrl (n = 6) rats, caused a sustained contracture which reached a plateau phase that persisted until washout. No statistical differences were observed in the amplitude of the direct muscular response to methacholine between the two animal groups (mean amplitude 1.1 ± 0.2 g and 1.2 ± 0.3 g for Ctrl and rWAS rats, respectively). Preparations from both OB (n = 14) and rWAS+OB (n = 18) groups exhibited the same motility pattern of the Ctrl (Figure 4Aa,c,d) and a similar amplitude of the GCs (Figure 4Ae).

### 3.5 TTX and L‐NNA effects on the spontaneous contractions

In strips from both Ctrl (n = 5) and Sham (n = 3) rats, addition of 1 × 10^‐6^ M TTX to the bath medium caused, after 10 minutes of contact time, a similar increase in amplitude of the spontaneous GCs (Figure 4B), thus indicating the removal of an inhibitory nervous control. Addition of 2 × 10^‐4^ M L‐NNA to the bath medium caused in both Ctrl (n = 5) and Sham (n = 3) rats an increase in amplitude of the spontaneous GCs (Figure 4B), suggesting the removal of an inhibitory control exerted by NO. The effects of L‐NNA were appreciable after 10‐15 minutes of contact time and persisted up to 1 hour (longer time not observed). The increase in amplitude of the spontaneous GCs caused either by TTX or L‐NNA was not statistically different between Ctrl and Sham animals, so the data were put and analysed together (Figure 4B). In preparations from rWAS rats, addition of TXX (n = 6) or L‐NNA (n = 6) to the bath medium greatly increased the amplitude of the small spontaneous contractions with respect to their own control (Figure 4B). Such increase was even major than that caused by the above drugs on the GCs of all the other animal groups (Figure 4B). Addition of TTX or L‐NNA to bath medium induced, in strips from both OB *(*n = 5 for each drug) and rWAS+OB (n = 6 for each drug) rats, an increase in amplitude of the spontaneous GCs not statistically different from that observed in Ctrl rats (Figure 4B).

Notably, in preparations from all the animal groups, the increase in amplitude of the spontaneous contractions caused by L‐NNA was greater than that elicited by TTX (Figure 4B).

### 3.6 Histology, histochemistry and immunohistochemistry

H&E staining (Figure S2) showed a substantial integrity of mucosa, submucosa, and muscle wall in all groups of animals. In the rWAS group, rare and spatially limited clusters of inflammatory cells were recognizable in the mucosa (Figure S2A,B). PAS staining (Figure S2) showed a mucous production similar in all groups of animals (Figure S2C‐E). Toluidine Blue staining (Figure S2) demonstrated an increased mucous acidity in rWAS rats compared to the other groups of animals (Figure S2F‐H).

c‐kit labelled mast cells, counted only in the mucosa, were increased in the rWAS rats (145.8 ± 13.88; n = 7) compared to Ctrl ones (119.4 ± 10.82; n = 7), but this difference did not reach the significance (*P* = .153).

**FIGURE 5:**
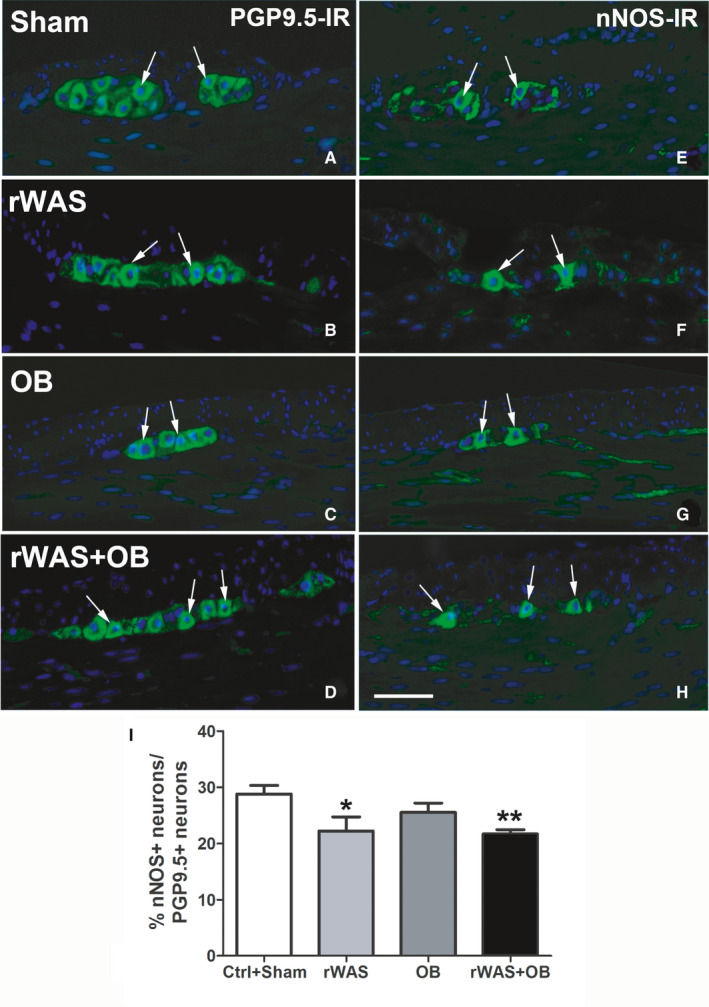
Protein Gene Product 9.5 (PGP9.5) and neuronal nitric oxide synthase (nNOS)‐immunoreactivity (IR). The PGP9.5 (A‐D) and nNOS (E‐H) labelling (green) was detected in the myenteric neurons and intramuscular nerve fibres of Sham (A and E), rWAS (B,F), OB (C and G) and rWAS+OB (D and H) rat distal colon. The Hoechst 33 342 labelled the nuclei (blue). The labelled neurons containing the nucleus were included in the statistical analysis. The arrows indicate the neurons that express both PGP9.5 and nNOS‐IR. Bar = 50 μm. Quantitative analysis of PGP9.5‐, nNOS‐ and iNOS‐IR neurons in the myenteric ganglia of rat distal colon (I). The values are expressed as percentage (%) of the total neurons labelled with the pan‐neuronal marker. The % of nNOS/PGP9.5‐IR myenteric neurons was significantly decreased in the rWAS and rWAS+OB rats compared to Ctrl+Sham and OB rats. One‐way ANOVA followed by Newman‐Keuls post‐test, *P < .05, **P < .005, n = 5

**FIGURE 6:**
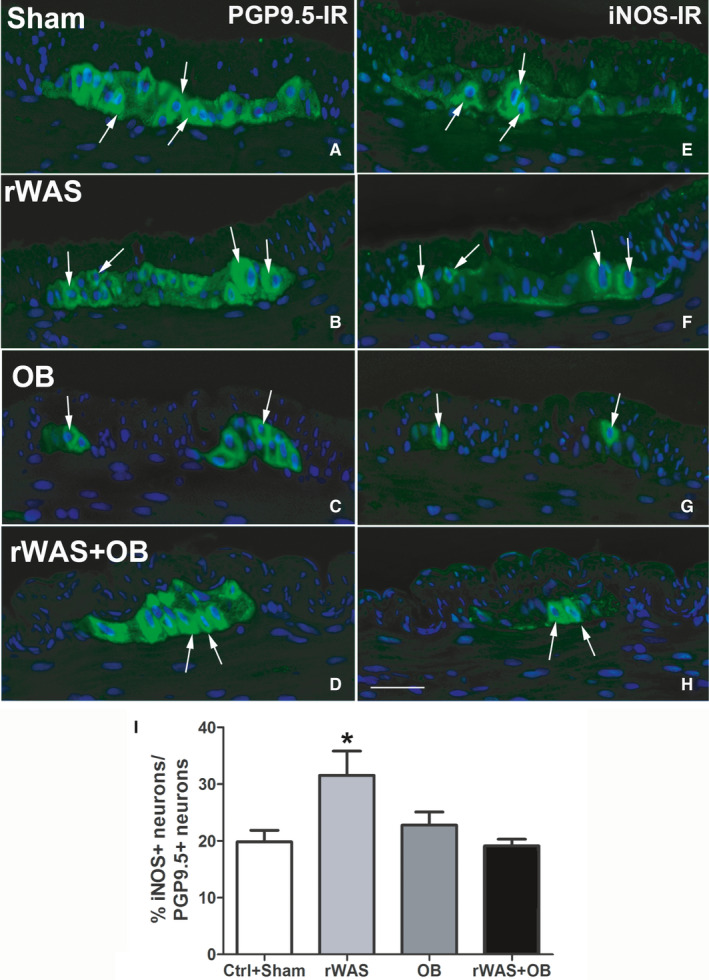
Protein Gene Product 9.5 (PGP9.5) and inducible nitric oxide synthase (iNOS)‐immunoreactivity (IR). The PGP9.5 (A‐D) and iNOS (E‐H) labelling (green) was detected in the myenteric neurons and intramuscular nerve fibres of Sham (A and E), rWAS (B and F), OB (C and G) and rWAS+OB (D and H) rat distal colon. The iNOS‐IR was faint, granular and mainly located in some myenteric neuronal bodies and intra‐ganglia nerve fibres. Rare intramuscular nerve fibres showed the labelling. The nuclei were counterstained in blue using Hoechst 33 342. The labelled neurons containing the nucleus were included in the statistical analysis. The arrows indicate the neurons that express both PGP9.5 and iNOS‐IR. Bar = 50 μm. Quantitative analysis of PGP9.5‐, nNOS‐ and iNOS‐IR neurons in the myenteric ganglia of rat distal colon (I). The values are expressed as percentage (%) of the total neurons labelled with the pan‐neuronal marker. The % of iNOS/PGP9.5‐IR myenteric neurons was significantly increased in the rWAS rats compared to all the other groups of rats. One‐way ANOVA followed by Newman‐Keuls post‐test, *P < .05, n = 5

Protein Gene Product 9.5 (PGP9.5) immunoreactivity (IR) was detected in the myenteric neurons and intramuscular nerve fibres of all groups of animals (Figures 5,6A‐D). Quantitation of the neurons displayed no significant differences among groups (Figures 5,6I).

neuronal nitric oxide synthase (nNOS)‐IR (Figure 5E‐H) and inducible nitric oxide synthase (iNOS)‐IR were observed in some myenteric neurons, and intramuscular nerve fibres and rare iNOS‐positive nerve fibres were seen in the inner portion of the circular muscle layer (Figure 6E‐H). nNOS‐positive neuron number expressed as percentage of the PGP9.5‐positive ones was significantly reduced in the rWAS and rWAS+OB rats respect to the other two groups (Figure 5I). This decrease was confirmed by WB analysis quantitation of the nNOS bands (Figure S3). Conversely, the number of iNOS‐positive neurons expressed as percentage of PGP9.5‐positive neurons was significantly increased in the rWAS rats respect to the other groups (Figure 6I). It was not possible to confirm this increase with WB due to the ineffectiveness of the antibody in detecting the expected band at the correct molecular weight on the membranes.

**FIGURE 7:**
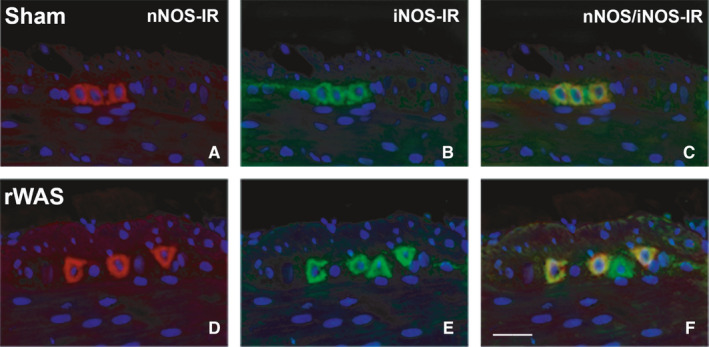
nNOS/iNOS double labelling. nNOS‐IR in red (A and D), iNOS in green (B and C); double labelling in orange (C and F) in the myenteric neurons of rat distal colon. Sham rats (A‐C): three neurons co‐expressing both labelling. rWAS rats (D‐F) three out of four iNOS‐IR neurons co‐expressed nNOS. Bar = 50 μm

nNOS/iNOS double labelled myenteric neurons were present in all rat groups. The two labelling were homogeneously distributed in the soma and in few myenteric fibres (Figure 7A‐F).

## 4 DISCUSSION

The present findings demonstrated the appropriateness of the rWAS rat model for studying IBS etio‐pathogenesis: daily exposure to one‐hour psychosocial stress for 10 consecutive days induced relevant behavioural and autonomic changes in the distal colon of these animals comparable to those observed in humans. Moreover, similarly to what happens when administered in IBS patients, oral OB treatment significantly counteracted some of these stress effects.

In our experimental conditions, rWAS showed face and construct validity in agreement with the criteria proposed by Meyer and Collins^31^ to validate an animal model. In fact, the simultaneous application of two relevant stressors, immobility and isolation, affected the rat behavioural response and the faecal output, and the results of the EPM after 10 days of stress disclosed a reduced exploratory performance that is a well‐established sign of anxiety in rodents.^32^ The increase in faecal production during the one‐hour stress indicates the involvement of the autonomic nervous system, likely through the activation of the brain‐gut axis,^8^, ^9^, ^33^ and attests to the presence of a colonic hyperactivity during stress exposition.^18^, ^34^ OB treatment did not avoid the development of stress‐like behaviour in rWAS rats, being this symptomatology generated in the brain; however, in rWAS+OB rats the drug progressively increased the stool consistency and significantly reduced the number of faecal pellets. An increased mucous acidity and a low‐grade inflammation were observed in the colonic mucosa of the rWAS rats. Changes in mucin quality were already described in similar animal models^35^ and related to dysbiosis.^36^ In IBS patients, although the mucous production has never been investigated, a dysbiosis with a frequency of 73%, compared to a rate of around 16% in healthy individuals has been reported.^37^ Dysbiosis increases intestinal permeability and induces low‐grade mucosal inflammation, conditions constantly present in IBS, which could favour visceral hypersensitivity and impair intestinal motility.^37^, ^38^ Accordingly, our rWAS rats showed an inflammatory picture, although mild, a mast cell increased number, although not significant, in the mucosa and an altered muscle contractility. These alterations were absent in the rWAS+OB rats suggesting that OB, possibly due to its bactericidal properties against pathogens as recently described ‘in vivo*’*,^27^ prevents mucous acidification and inflammation. Combining functional and morphological studies, we confirm the presence of colonic hyperactivity during the application of stress^34^, ^39^ but also found an unexpected, marked reduction of the spontaneous contractions of the circular muscle strips at the end of the stressful period associated with an increase of the myenteric iNOS‐IR neurons and a decrease of the nNOS‐IR ones. The motor pattern recorded in strips from Ctrl and OB rats, consisting of high‐amplitude contractions (CGs) superimposed on small‐amplitude oscillations, agrees with that described by Bayer et al.^40^ However, in rWAS strips, this pattern was deeply changed showing a depression of the spontaneous motility and absence of the GCs. These changes were not ascribable to a reduced muscle responsiveness, since the magnitude of the response to methacholine in Ctrl and rWAS rats was comparable. Even if the reduction of the spontaneous motility observed in rWAS preparations was surprising, other studies reported a consistent slowed colonic motility during inflammatory bowel disease frequently accompanied by symptoms of diarrhoea, both in humans and in animal models of colitis.^41^, ^42^, ^43^ Noteworthy, TTX and L‐NNA increased the amplitude of spontaneous contractions indicating the removal of an inhibitory control attributable, at least in part, to nitric oxide (NO). In fact, NO, considered the main physiological modulator of intestinal motility, plays an important role in controlling the amplitude of GCs.^44^ L‐NNA effects were greater than those of TTX, suggesting the involvement of a not‐neurogenic (myogenic?) NO production as well. Indeed, we cannot establish if and how much NO of neuronal origin participates in the TTX‐induced increase amplitude of the spontaneous contractions since other inhibitory neurotransmitters could be involved. On the other hand, we showed significant changes in the nNOS‐ and iNOS‐positive myenteric neurons in rWAS rats. The presence of iNOS‐positive neurons in normal conditions is not surprising,^30^, ^45^ and, presently, we showed that the iNOS‐positive neurons were the 20% of the total myenteric neurons while the nNOS‐positive the 30%; besides almost all the iNOS‐labelled neurons also expressed nNOS. Intriguingly, in the rWAS rats after 10 days of treatment the nNOS‐positive neurons were significantly decreased and the iNOS ones increased. Contrasting results are present in the literature related to nNOS expression in the colon of IBS animal models,^16^, ^19^ while no data are available on iNOS neuronal expression either in IBS or in IBS animal models. Although the present nNOS decrease apparently conflicts with our mechanical data in rWAS rats, the significant increase of the iNOS‐IR neurons well supports the depressed motility pattern observed in these animals. It is interesting to note that in rWAS rats the proportion of nNOS to iNOS‐positive neurons was reversed compared to controls and this datum allows us to hypothesize that the increase in iNOS‐IR occurred in the proportion of nNOS‐positive neurons that did not have expressed (at least at detectable levels) the inducible isoform under control conditions. If so, we could assume that the decrease in nNOS expression depends on the increase in the inducible isoform, as it is known that the high concentrations of NO produced by the iNOS hinder the transcription of the constitutive NOS gene.^46^ A high production of NO due to iNOS also explains the greater effects of TTX and L‐NNA in rWAS than in the other groups (when expressed as a percentage of their own control) indicating a stronger inhibitory control in the stressed rats.

Commonly, high NO levels are present in inflammatory gastrointestinal status such as Crohn's disease, ulcerative colitis^47^ or coeliac disease^48^ and mainly attributable to an immune cell or glial cell production. However, as observed in our rWAS rats, an increase of NO was also found in IBS patients which did not present an important inflammatory picture.^49^ Thus, in the present work it is shown, for the first time in an animal model of IBS, that myenteric neurons might be a significant source of NO in the absence of consistent inflammation. However, although our mechanical results show that the effects caused by stress are mainly presynaptic, being TTX‐sensitive, a postsynaptic involvement cannot be ruled out. Indeed, a nNOS myogenic splice variant as well as an inducible isoform in the rodent smooth muscle cells have been identified.^30^, ^50^, ^51^, ^52^ Unfortunately, despite the numerous attempts made, we cannot confirm this datum due to the absence of reliable antibodies labelling the diverse NOS isoforms.

Interpreting the quantitative changes of the two NOS isoforms in rWAS rats is not obvious. Possibly, we must consider the role of the hypothalamic corticotropin‐releasing hormone (CRF), primary mediator of the psychosocial stress in mammals.^53^ CRF injection in rodents triggers adverse intestinal effects mediated by the CRF1 and CRF2 receptors mimicking those produced by the stress.^33^, ^34^, ^54^ CRF binds to both receptor types with higher affinity for the CRF1r.^34^, ^55^ CRF1r activation causes the adverse effects, while CRF2r, that ‘per se*’* has no effects, hampers CRF1r activation. In the rat distal colon, almost 95% of the myenteric neurons express CRF1r and, when CRF bound to the receptor, in more than 50% of them *c‐fos* expression significantly increases^34^, ^55^, ^56^ accompanied by an augmented defecation.^55^ Moreover, 80% of the neurons expressing *c‐fos* are nitrergic^55^ and the neuronal *c‐fos* activation promotes the transcription of neurotransmitter biosynthetic enzymes.^57^ Based on all these data, we hypothesize that the changes of the two NOS isoforms detected in rWAS rats result from the promotion of the iNOS (rather than nNOS) gene transcription in the myenteric neurons through CRF1r‐*c‐fos* activation. The occurrence of a CRF1r‐*c‐fos* activation can also help to explain the beneficial effects of OB treatment. It has been reported in a rat colitis model that OB significantly reduced *c‐fos* expression in lumbosacral spinal cord neurons^58^ and that chronic OB administration in rats subjected to a psychosocial stress prevented the CRF1r increase in the colonic myenteric neurons without affecting the simultaneous CRF2r increase.^14^, ^59^ Thus, we propose that OB, preventing CRF1r activation, interrupts the cascade events that bring to the mechanical and immunohistochemical changes presently described in rWAS rats. The finding that OB did not affect the CFR2r increase in a model of psychosocial stress^14^ might be considered ‘per se*’* a condition to hinder this cascade. In agreement with this possibility, Sweetser et al^60^ attributed the lack of efficacy of the CRF1r antagonist to improve colonic symptoms in patients with IBS to an impairment of the integrated CRF1r‐CRF2r response.

In conclusion, the present study confirms rWAS as a reliable animal model to investigate the cellular mechanisms responsible for IBS. By combining functional and morphological experiments, we demonstrate how stress alters distal colonic motility by enhancing the inhibition of the muscle contractility through an increased expression of iNOS in myenteric neurons. This increase as well as the changes in the motor pattern are prevented by oral administration of OB.

## CONFLICT OF INTEREST

The authors declare no conflicts of interest.

## AUTHOR CONTRIBUTION

**Chiara Traini:** Conceptualization (supporting); Data curation (equal); Formal analysis (equal); Investigation (lead); Methodology (equal). **Eglantina Idrizaj:** Data curation (equal); Formal analysis (equal); Investigation (equal); Methodology (equal); Project administration (supporting). **Rachele Garella:** Data curation (supporting); Investigation (equal). **Maria‐Simonetta Faussone‐Pellegrini:** Conceptualization (supporting); Data curation (equal); Formal analysis (equal). **Maria Caterina Baccari:** Conceptualization (supporting); Data curation (equal); Formal analysis (equal); Investigation (equal); Methodology (equal); Project administration (supporting). **Maria Giuliana Vannucchi:** Conceptualization (lead); Data curation (equal); Formal analysis (equal); Funding acquisition (lead); Project administration (lead).

## Supporting information

Figure S1

Figure S2

Figure S3

Table S1

## Data Availability

Data available on request from the authors.
