# Supplementary figures and images for: Otilonium Bromide treatment prevents nitrergic functional and morphological changes caused by chronic stress in the distal colon of a rat IBS model
Source: J Cell Mol Med. 2021 Jun 9;25(14):6988–7000. doi: 10.1111/jcmm.16710 (PMC8278105; doi:10.1111/jcmm.16710)

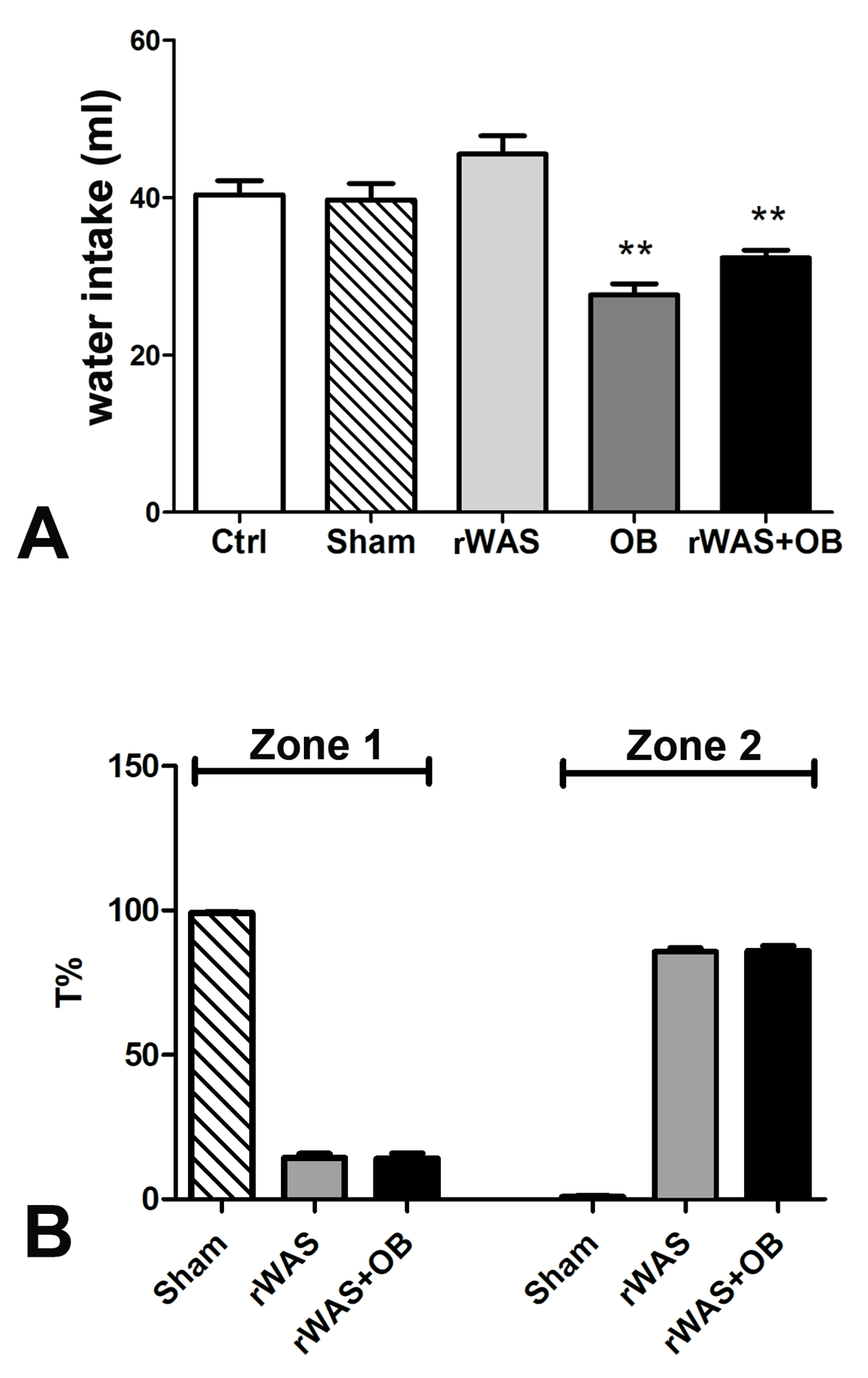

Supplement: Supplementary file 1 — Figure S1 [file JCMM-25-6988-s001.tif]

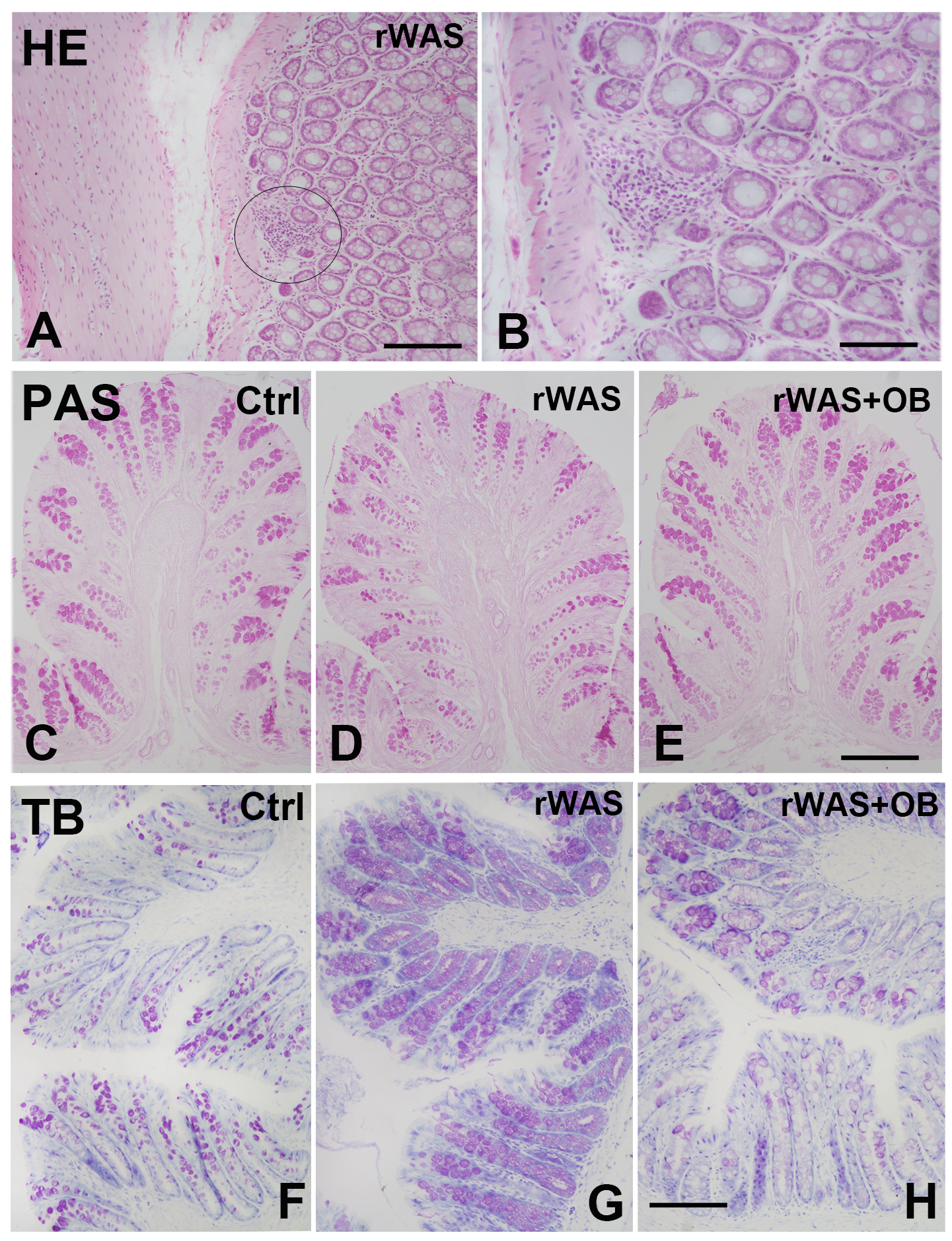

Supplement: Supplementary file 2 — Figure S2 [file JCMM-25-6988-s002.tif]

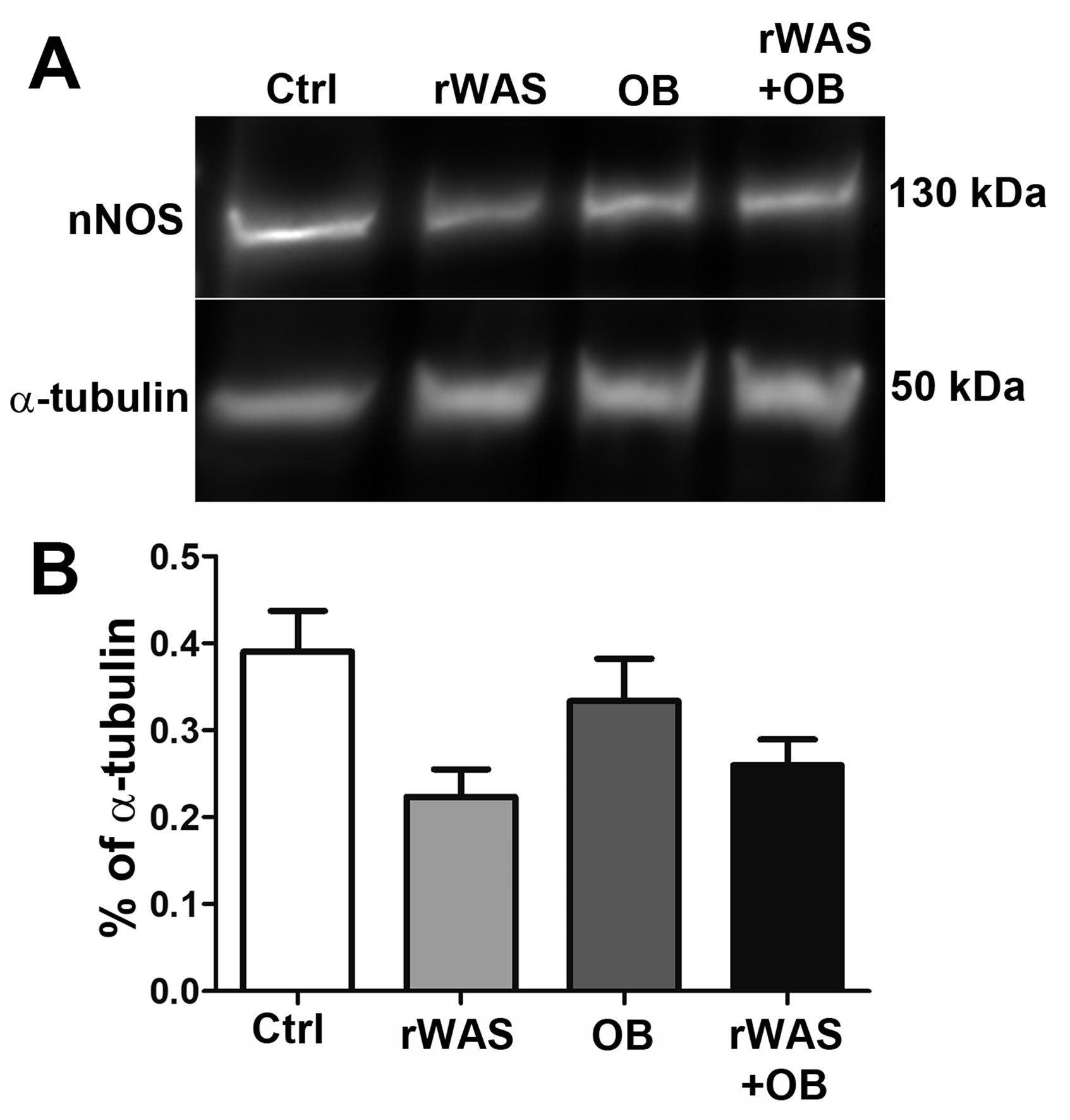

Supplement: Supplementary file 3 — Figure S3 [file JCMM-25-6988-s003.tif]
