# Supplementary material for: Otilonium Bromide treatment prevents nitrergic functional and morphological changes caused by chronic stress in the distal colon of a rat IBS model
Source: J Cell Mol Med. 2021 Jun 9;25(14):6988–7000. doi: 10.1111/jcmm.16710 (PMC8278105; doi:10.1111/jcmm.16710)
Supplement: Supplementary file 4 — Table S1 [file JCMM-25-6988-s004.docx]

| Primary antibody | **Host** | **IHC WB** | **Producer** |
| --- | --- | --- | --- |
| anti-PGP9.5 | Rabbit | 1:200 | AB1761-I; EMD Millipore Corporation,  Temecula, CA, USA |
| anti-nNOS | Rabbit | 1:2000 1:2500 | AB5380; EMD Millipore Corporation |
| anti-nNOS | Mouse | 1:250 | BD Transduction Laboratories. Lexington, KY, USA |
| anti-iNOS | Rabbit | 1:400 1:2500 | 482728; Calbiochem; EMD Millipore  Corporation |
| anti-iNOS | Mouse | 1:50 1:500 | sc-7271; Santa Cruz Biotechnology,  Santa Cruz, CA, USA |
| anti-c-kit | Rabbit | 1:300 | A4502; Dako, Glostrup, Denmark |
| anti-α-tubulin | Mouse | 1:5000 | T6074 Sigma-Aldrich, Merck Life Science S.r.l., Milano, Italy |
| anti-β-actin | Rabbit | 1:20000 | A2066; Sigma-Aldrich, St. Louis, MO,  USA |
| **Secondary antibody** | **Host** | **IHC** | **Producer** |
| anti-rabbit | Goat | 1:333 | Invitrogen, San Diego, CA, USA |
| anti-rabbit | Goat | 1.15000 | Jackson Immuno Research labs, Baltimora PA, USA |
| anti-mouse | Goat | 1:15000 | Jackson ImmunoReasearch Labs |

**Table S1. List of primary and secondary antibodies**
